# Fourth Annual Conference of the Council on Medical Education of the American Medical Association Held at Chicago, April 13, 1908

**Published:** 1908-05

**Authors:** 


**BUFFALO MEDICAL JOURNAL.**

**A Monthly Review of Medicine and Surgery.**

*EDITOR:*

WILLIAM WARREN POTTER, M. D.

All communications, whether of a literary or business nature, books for review and exchanges, should be addressed to the editor 238 DELAWARE AVE., BUFFALO, N. Y.

VOL. LXIII. MAY, 1908. No. 10

### Fourth Annual Conference of the Council on Medical Education of the American Medical Association held at Chicago, April 13, 1908.

THE growing interest in these conferences was manifest by the large attendance of members and delegates from all parts of the country. The meeting was called to order at ten o'clock A. M., and the chairman, Arthur Dean Bevan, immediately delivered his address, which dealt chiefly with progress made in medical standards during the year. Attention was invited to the merging of medical colleges in Minnesota and Indiana, commending the action and urging similar procedures in other regions.

The secretary, N. P. Colwell, then read his report which was illustrated by six colored maps, showing the status of medical education and reciprocity in medical licensure throughout the United States. These maps are of special interest and will no doubt be studied by all educators when they appear in the report of the Council.

The report of the committee on preliminary education was presented by John H. Long, Chairman. This was discussed by Charles W. Eliot, President of Harvard University, who spoke at some length in urging high standards of preliminary education, calling attention to the fact that colleges requiring such standards for admission are more apt to receive endowments. He said, contrary to the general belief, that more than one-half the endowments of Harvard had come from outside New England, largely from New York. President Eliot's remarks were received with marked attention and he was accorded hearty applause. Charles S. Sheldon, of Madison, Wis., the delegate from the American Academy of Medicine, next discussed the report and during the course of his remarks expressed the opinion that work in physics, chemistry and biology could not be completed properly in less than two years of university time. The next speaker was A. H. Freeland Barbour, professor of gynecology in the University of Edinburgh, who by invitation gave the requirements governing medical education in Scotland. Professor Barbour, now on a visit to this country, was most opportunely present at the Conference and his remarks added conspicuous interest to the occasion.

Victor C. Vaughan, chairman, then read the report of the committee on "What should constitute a Medical College in good standing." James W. Holland, Dean of the Jefferson Medical College, Philadelphia, made an elaborate speech in discussing this question, the trend of which was, while advocating reasonably high standards, changes should be made prudently and with slow haste lest prevailing conditions be upset too suddenly. He repeatedly complimented the State of New York on the stand it has taken relating to medical education.

At the afternoon session the committee on Essentials of a Model Medical Practice Act reported through its chairman, Beverly D. Harrison, secretary of the Michigan State Board of Registration. The report was discussed by F. Dudley Tait, San Francisco, and William Warren Potter, Buffalo, the other members of the committee. Dr. Tait presented an elaborate analysis of the subject, while Dr. Potter accentuated one point—namely, that of time. He suggested that any practice law should be constructed with a view to economising time, which is such an essential element in these days of such rapidity of movement. The subject was further discussed by Alexander R. Craig of the Pennsylvania State Medical Society.

The special discussions on the "Character of the State Medical Licensing Examination" and on "Practical Ideas Concerning Reciprocity" were considered together. A. Ravogli, President of the Ohio State Board of Medical Education, and S. D. Van Meter, secretary of the State Board of Medical Examiners of Colorado, were the principals in the discussion on practical ideas concerning reciprocity. J. W. Bennet, secretary of the State Board of New Jersey, W. T. Councilman, professor of pathology at Harvard University Medical School, F. F. Wesbrook, Dean of College of Medicine and Surgery of the University of Minnesota, and J. A. Witherspoon, professor of medicine at Vanderbilt University Medical department, were the principal speakers in the discussion on Character of the State Medical Licensing Examination." Dr. F. J. Lutz, of Saint Louis, also spoke interestingly on the subject. The general opinion seemed to be that this examination should be fashioned so as to discriminate between the candidate trained in laboratories and clinics and one who has only a "quiz compend" or theoretical education.

Dr. Herbert L. Burrell, of Boston, President of the American Medical Association, attended the Conference by invitation and was present during both sessions, manifesting great interest in the proceedings.

An interesting feature of the secretary's report was the statement that 34 states now have reciprocal relations with 4 or more other states; that during the past year 834 licenses were issued through reciprocity—an increase of 149 over 1906.

About 100 delegates were present and the Conference was held in session until 6.30 P. M.

---

THE city of Syracuse was much smaller than Buffalo when its Common Council voted $100,000 as a gift, to induce Genesee College of Lima to move to that city and become a part of Syracuse University. This example should serve as a stimulus to Buffalo to contribute liberally from its municipal exchequer to promote the establishment of a Greater University in this fair city. The example of Syracuse should serve, likewise, as a rebuke to the board of supervisors of the County of Erie for its illiberal, not to say parsimonious, method of dealing with the question last winter. The enterprise of this great city in engaging in large commercial activities should be supplemented by a similar spirit in educational affairs. It is a disgrace for it to continue to manifest apathy in so important a question, one involving such large interests to its young people.

---

THE employment of trained women as nurses in the United States navy would seem to be approaching a realisation. A bill providing for the establishment of such a corps has been reported favorably to the house of representatives by the committee on naval affairs. The bill provides for a superintendent nurse, appointed by the Secretary of the Navy, who shall be in direct charge of the corps, and responsible for their organisation and discipline. She will keep in touch with the training schools for nurses and with the standing of their graduates, having in view the efficient and proper recruitment of the corps, pass upon the general fitness of applicants and prescribe examinations, look after the professional efficiency of the nurses after entering the corps as well as to keep the duty rosters and efficiency records.

The chief nurses will be assigned to duty in each of the larger naval hospitals. All nurses must be graduated from a hospital training school, having a course of not less than two years. They may be detailed to duty on board of hospital and ambulance ships and for special duty by the Surgeon General of the Navy, under whom the corps is organised.

"Their special fitness for hospital work is universally recognised," says the report in speaking of the proposed corps of female nurses, "and this bill would put nursing in naval hospitals on a professional plane, in keeping with the efficiency which prevails generally." The nurses shall receive the same pay as that for the same corps in the army, where the superintendent receives $1,800.00 per annum, and nurses and reserve nurses on active service $40.00 per month on duty in the United States, and $50.00 per month when without the limits of the United States. The army has 100 female nurses, but the Surgeon General of the Navy, believes that 50 will be sufficient for the present needs of the navy. Secretary Metcalf and Surgeon General Rixey have recommended the organisation of this corps.

---

The city of Niagara Falls is to be congratulated upon having taken the first step toward securing pure water for the use of its inhabitants and visitors. Without a dissenting vote, the common council of the Cataract City, on April 17, 1908, approved the compromise charter amendment, providing for the appointment by the mayor of a water commission of six members, to be confirmed by the council. The result, so utterly unexpected, was greeted with cheers and applause.

The charter amendment now goes to the mayor and then to the Governor for final approval. It gives to the commission created by it full power to consider plans, hire engineers, receive specifications and, in fact, do all things necessary to get the matter into definite shape for submission to the taxpayers. The commissioners are to serve without pay.

The pollution of the waters of Niagara river has long been a disgrace for which Buffalo has been responsible in large measure, and it is commendable that Niagara Falls should take the initiative toward ridding itself of this menace to health on Good Friday in this year of grace.

